# High sperm DNA fragmentation is associated with increased embryonic chromosomal abnormality: a retrospective study of 8 years involving 5637 couples undergoing ART

**DOI:** 10.1093/hropen/hoag082

**Published:** 2026-09-16

**Authors:** Li Lou, Pengcheng Xu, Pingyuan Xie, Huan Zhang, Yifan Gu, Fei Gong, Ge Lin, Guangxiu Lu, Jing Dai, Shujuan Ma

**Affiliations:** NHC Key Laboratory of Human Stem Cell and Reproductive Engineering, Xiangya School of Basic Medical Sciences, Central South University, Changsha, China; School of Public Health, Hunan Normal University, Changsha, China; Health Science Center, Hunan Normal University, Changsha, China; NHC Key Laboratory of Human Stem Cell and Reproductive Engineering, Xiangya School of Basic Medical Sciences, Central South University, Changsha, China; Clinical Research Center for Reproduction and Genetics in Hunan Province, Reproductive & Genetic Hospital of CITIC-Xiangya, Changsha, China; NHC Key Laboratory of Human Stem Cell and Reproductive Engineering, Xiangya School of Basic Medical Sciences, Central South University, Changsha, China; Clinical Research Center for Reproduction and Genetics in Hunan Province, Reproductive & Genetic Hospital of CITIC-Xiangya, Changsha, China; NHC Key Laboratory of Human Stem Cell and Reproductive Engineering, Xiangya School of Basic Medical Sciences, Central South University, Changsha, China; Clinical Research Center for Reproduction and Genetics in Hunan Province, Reproductive & Genetic Hospital of CITIC-Xiangya, Changsha, China; NHC Key Laboratory of Human Stem Cell and Reproductive Engineering, Xiangya School of Basic Medical Sciences, Central South University, Changsha, China; Clinical Research Center for Reproduction and Genetics in Hunan Province, Reproductive & Genetic Hospital of CITIC-Xiangya, Changsha, China; NHC Key Laboratory of Human Stem Cell and Reproductive Engineering, Xiangya School of Basic Medical Sciences, Central South University, Changsha, China; Clinical Research Center for Reproduction and Genetics in Hunan Province, Reproductive & Genetic Hospital of CITIC-Xiangya, Changsha, China; NHC Key Laboratory of Human Stem Cell and Reproductive Engineering, Xiangya School of Basic Medical Sciences, Central South University, Changsha, China; Clinical Research Center for Reproduction and Genetics in Hunan Province, Reproductive & Genetic Hospital of CITIC-Xiangya, Changsha, China; Clinical Research Center for Reproduction and Genetics in Hunan Province, Reproductive & Genetic Hospital of CITIC-Xiangya, Changsha, China

**Keywords:** sperm DNA integrity, DFI, PGT-A, early embryonic development, aneuploidy

## Abstract

**STUDY QUESTION:**

Does sperm DNA fragmentation impair blastocyst chromosomal integrity?

**SUMMARY ANSWER:**

A high sperm DNA fragmentation index (DFI) is associated with an increased risk of blastocyst aneuploidy, particularly when the man has poor semen quality or when the female partner is aged ≥35 years.

**WHAT IS KNOWN ALREADY:**

Evidence linking sperm DFI to embryo development and blastocyst chromosomal integrity remains inconsistent, and controversy persists regarding its association with blastocyst aneuploidy detected by preimplantation genetic testing for aneuploidy (PGT-A). Existing studies have often been limited by small sample sizes, inconsistent DFI assays, and insufficient controls of key confounders.

**STUDY DESIGN, SIZE, DURATION:**

This retrospective cohort study was conducted at a tertiary reproductive center. A total of 5637 infertile couples undergoing assisted reproduction between January 2016 and March 2023 were screened, and 3948 couples with complete data were included after applying standardized exclusion criteria.

**PARTICIPANTS/MATERIALS, SETTING, METHODS:**

All male partners who underwent sperm DNA fragmentation assessment using the Sperm Chromatin Structure Assay were included. Infertile couples were stratified by treatment modality, age of both partners, and male semen quality parameters. The primary outcome was blastocyst aneuploidy rate. Beta-binomial regression and zero-inflated beta-binomial regression were used to analyze embryonic outcomes, with model selection guided by the Akaike Information Criterion. Generalized linear mixed models were applied to account for clustering of multiple embryos within the same ovarian stimulation cycle.

**MAIN RESULTS AND THE ROLE OF CHANCE:**

Higher DFI was associated with a higher rate of blastocyst aneuploidy. Compared to the low DFI group, the high DFI group had a significantly higher adjusted odds ratio (aOR) of aneuploidy (38.19% vs 24.42%, aOR = 1.35, 95% CI: 1.10–1.81, *P *= 0.042), while there was no substantial difference between the medium and low DFI group (25.78% vs 24.42%, aOR = 0.93, 95% CI: 0.76–1.13, *P *= 0.465). Particularly, when women were aged ≥35 years, the embryonic aneuploidy rate in the high DFI group was significantly higher than low DFI subgroup (52.02% vs 38.75%, aOR = 1.61, 95% CI: 1.12–2.32, *P *= 0.010). Also, among men with severe oligoasthenoteratozoospermia, high DFI significantly increased aneuploidy risk (36.81% vs 18.03%, aOR = 1.99, 95% CI: 1.11–3.57, *P *= 0.022). For all men aged <40 years, high DFI was associated with a reduced likelihood of high-quality blastocyst formation (aOR = 0.79, 95% CI: 0.64–0.99, *P *= 0.044).

**LIMITATIONS, REASONS FOR CAUTION:**

Given the single-center retrospective design, residual confounding cannot be excluded despite rigorous statistical adjustment.

**WIDER IMPLICATIONS OF THE FINDINGS:**

Routine sperm DFI assessment may provide clinically meaningful information for infertile couples, particularly those with advanced maternal age or compromised male fertility. These findings support the integration of DFI into embryo risk stratification and individualized treatment planning, potentially improving reproductive outcomes and resource allocation.

**FUNDING:**

This work was supported by the National Natural Science Foundation for Young Scientists (72504291), Hunan Provincial Natural Science Foundation of China (2025JJ50703), and the Scientific Research Foundation of Reproductive and Genetic Hospital of CITIC-Xiangya (YNXM-202411).

**DISCLOSURES:**

None of the authors have any conflicts of interest to disclose.

**TRIAL REGISTRATION NUMBER:**

N/A.

WHAT DOES THIS MEAN FOR PATIENTS? When a couple is having difficulty achieving pregnancy, doctors often check the man’s sperm concentration and motility. However, even when these basic tests look normal, the genetic material inside the sperm (its DNA) can still be damaged. Whether this hidden damage increases the risk of chromosomal abnormality in embryos has been uncertain.

Our study looked at 8 years of clinical data from 5637 couples undergoing fertility treatment followed by preimplantation genetic testing of the embryos for chromosomal abnormalities: i.e. aneuploidy (an incorrect number of chromosomes). We found that higher levels of DNA damage in sperm were associated with a greater chance of aneuploidy in embryos. This risk was enhanced in couples with advanced maternal age or poor semen quality.

These findings suggest that testing for sperm DNA damage before treatment could help doctors better understand a couple’s chances of producing healthy embryos. This information can support more personalized treatment planning and help couples make informed decisions about their fertility care.

## Introduction

The global incidence of male infertility is rising, with ART serving as a primary treatment option (Agarwal *et al.*, 2021). Embryo quality is a critical determinant of success, and as the carrier of the paternal genome, sperm play an essential role in embryonic development. However, conventional semen parameters fail to detect functional defects, prompting greater reliance on sperm DNA fragmentation (SDF) testing to assess chromatin quality (Agarwal and Allamaneni, 2005). The DNA fragmentation index (DFI), a quantitative measure of SDF, has become a critical biomarker of sperm nuclear integrity (Agarwal *et al.*, 2020) and is increasingly recognized as a valuable adjunct to conventional semen analysis. Among DFI assays, the Sperm Chromatin Structure Assay (SCSA) is widely regarded as a standardized clinical tool due to its high reproducibility, automated analysis, and established clinical thresholds (Bungum *et al.*, 2007; Evenson *et al.*, 2020; Behdarvandian *et al.*, 2023).

Although the impact of sperm DFI on assisted reproduction outcomes has been widely investigated, the evidence remains contentious. While some studies associate elevated DFI with impaired fertilization, compromised embryo quality, decreased pregnancy rate (Simon *et al.*, 2014; Zhao *et al.*, 2014; Jiang *et al.*, 2025), and recurrent pregnancy loss (McQueen *et al.*, 2019; Yuan *et al.*, 2019), others report no substantial correlation with ART success rates (Liu *et al.*, 2023; Fang *et al.*, 2025; Wang *et al.*, 2026).

Controversy is particularly pronounced regarding blastocyst chromosomal integrity assessed via preimplantation genetic testing for aneuploidy (PGT-A), with conflicting findings ranging from positive associations (Fu *et al.*, 2023; Gao *et al*., 2023; Ping *et al.*, 2023) to null effects in oocyte donation models (Gat *et al.*, 2018). These inconsistencies are largely attributable to methodological shortcomings, including small sample sizes, variability in DFI assessment, and inadequate control for key confounders.

To address these critical gaps, we conducted a large-scale retrospective cohort study to systematically evaluate the impact of DFI on early embryo developmental competence and blastocyst chromosomal integrity. This study aims to provide clinical evidence to guide the integration of DFI assessment into male infertility evaluation and individualized ART treatment strategies.

## Materials and methods

### Study design and participants

This retrospective cohort study was based on data from the CITIC-Xiangya Assisted Reproductive Technology (CXART) cohort (Ma *et al.*, 2024). The CXART cohort is registered on ClinicalTrials.gov (NCT05404464). It is an ongoing, prospectively maintained, longitudinal registry established at the Reproductive and Genetic Hospital of CITIC-Xiangya in Changsha, China. It systematically collects multidimensional clinical, laboratory, embryological, and long-term follow-up data from patients undergoing ART treatments. For the present analysis, we screened 5637 ART cycles performed between 1 January 2016 and 31 March 2023. Eligible cycles included couples undergoing their first ART attempt, where the male partner had completed SCSA and conventional semen analysis, the female was aged 20–45 years, and couples possessed normal chromosomal karyotypes. Exclusion criteria comprised cycles involving *in vitro* maturation (IVM), preimplantation genetic testing for monogenic diseases (PGT-M) or structural rearrangements (PGT-SR), oocyte or sperm donation, frozen semen samples, or an interval exceeding 1 year between DFI assay and ART treatment. This study was approved by the Ethics Committee of the Reproductive and Genetic Hospital of CITIC-Xiangya (Ethics Number: LL-SC-2025-019), and the requirement for informed consent was waived due to the retrospective nature of the study.

### Semen detection

Semen analysis was performed according to the WHO Laboratory Manual for the Examination and Processing of Human Semen (6th edition; World Health Organization, 2021). After 2–6 days of sexual abstinence, semen samples were collected by masturbatory ejaculation at the hospital, which were then fully liquefied in a 37°C incubator. After liquefaction for 30 min, sperm concentration and motility were assessed manually or using Computer-Aided Sperm Analysis (CASA) (Davis and Katz, 1993). Semen processing was performed in a standardized manner according to the routine clinical protocol of our center, using either density gradient centrifugation or the swim-up method; microfluidic-based sperm selection was not used in our cohort. Sperm morphology was evaluated according to WHO standards. Severe oligoasthenoteratozoospermia (SOAT) was defined as a sperm concentration <5 million/ml, a progressive motility rate <10%, or a normal sperm morphology rate <2%.

### DFI assay

The sperm DFI assay was analyzed using SCSA on the original semen sample (Behdarvandian *et al.*, 2023). Sperm samples were treated with a low-pH detergent solution (containing 0.01% Triton X-100, 0.15 M NaCl, and 0.08 N HCl; pH 1.4) for 30 s, followed by staining with 6 mg/l acridine orange (AO) solution (Topregmed, Chengdu, China) in phosphate-citrate buffer (pH 6.0). Analysis was performed using a BD FACSC alibur flow cytometer (Becton Dickinson, Franklin Lakes, NJ, USA) equipped with an air-cooled argon ion laser (tuned to 488 nm). The flow rate of sperm during detection was set to 100–300 cells per second; data collection commenced 2 min after initiation, with a minimum of 5000 cells analyzed. When excited by a blue light source, AO intercalated with intact DNA emitted green fluorescence (530 ± 30 nm), while AO bound to damaged DNA emitted red fluorescence (>630 nm). Results were presented in a cytogram pattern; the extent of sperm chromatin damage was quantified by evaluating the intensity levels of green (indicating native double-stranded DNA) and red (indicating denatured single-stranded DNA) fluorescence.

### Embryo assessment and main outcomes

Embryos were cultured to cleavage stage (Day 3) or to blastocyst stage (Days 5–6). Embryo transfer, limited to one or two embryos per cycle, was performed based on morphological evaluation. Primary outcomes were the high-quality blastocyst rate and the incidence of chromosomal abnormalities. All cleavage-stage embryo assessments were performed using the Istanbul Consensus (Coticchio *et al.*, 2025), and blastocyst quality assessments were performed using the Gardner scoring system (Gardner *et al.*, 2019). Cleavage-stage embryos with more than six cells and <20% fragmentation were regarded as high-quality embryos. Blastocysts graded ≥4BB were regarded as high-quality blastocysts. In accordance with our routine clinical protocol, trophectoderm biopsy was performed preferentially on the four highest-grade blastocysts available per patient during the initial testing, with grading based on morphological criteria. Additional biopsies will be conducted on the remaining embryos as appropriate. Among the blastocysts analyzed by PGT-A, chromosomal abnormalities were categorized as: aneuploidy (chromosomal numerical abnormalities), mosaicism (abnormal cell proportion between 20% and 80%), and segmental abnormalities (segmental deletions/duplications ≥4.0 Mb). Secondary outcomes included the following rates: fertilization rate (total fertilized oocytes/retrieved oocytes), normal fertilization rate (two-pronuclear zygotes/retrieved oocytes), cleavage rate (embryos reaching the two-cell stage or above/total fertilized oocytes), high-quality embryo rate (embryos more than six cells and <20% fragmentation on Day 3/total fertilized oocytes), blastocyst rate (total blastocysts/cultured embryos), and high-quality blastocyst rate (blastocysts graded ≥4BB/cultured embryos).

### Statistical analysis

The overall statistical strategy comprised three core components: (i) characterization of DFI as the primary exposure, (ii) modeling of embryo developmental parameters, and (iii) targeted analyses of PGT-A outcomes. Continuous variables are presented as medians (interquartile range) and categorical variables as frequencies (percentages). Group comparisons were performed using the Kruskal–Wallis test or one-way ANOVA for continuous variables and the Chi-square test for categorical variables, as appropriate.

DFI was analyzed both as a continuous and a categorical variable. When treated as a continuous variable, restricted cubic spline functions were applied to explore linear and nonlinear associations between DFI and conventional semen parameters, as well as embryo development parameters. For categorical analyses, DFI was stratified according to existing clinical thresholds into three groups: <15% (low), 15–30% (medium), and ≥30% (high) (Virro *et al.*, 2004; Lin *et al.*, 2008). This categorized DFI variable was treated as a continuous term to calculate the *P* for trend.

Embryo development parameters, including fertilization rate, high-quality embryo rate, and blastocyst formation rate, were analyzed using beta-binomial or zero-inflated beta-binomial regression, which accounted for the proportional structure, overdispersion, and potential excess zeros of the data. Model selection was guided by the Akaike Information Criterion. For PGT-A outcomes, generalized linear mixed models (GLMMs) were employed to account for the non-independence of multiple embryos derived from the same oocyte retrieval cycle (random intercept for patient‑cycle identification number (PID)).

Multivariable models were adjusted for clinically relevant factors, including male age, male smoking status, female age, female BMI, female smoking status, polyendocrine metabolic ovarian syndrome (PMOS), SOAT, fertilization method (IVF vs ICSI), and blastocyst quality (high-quality vs non-high-quality), in line with previously published literature (Coticchio *et al.*, 2025). Covariates were selectively applied based on each specific outcome under investigation.

To assess robustness, two sensitivity analyses were conducted. First, propensity score overlap weighting (OW) was applied to balance baseline characteristics across DFI categories. Second, the primary analyses were repeated after restricting the cohort to cycles in which DFI testing was performed within 6 months prior to oocyte retrieval. Pre-specified subgroup analyses were conducted to assess the association between DFI levels and embryological or PGT-A outcomes across key demographic and clinical strata, including female age (<35 vs ≥35 years), male age (<40 vs ≥40 years), SOAT status (yes vs no), fertilization method (IVF vs ICSI), and high-quality blastocyst morphology (yes vs no). To formally evaluate potential effect modification, interaction terms between each subgroup variable and categorized DFI were incorporated into the respective regression models, and the corresponding *P* values for interaction were reported. A two-sided *P* value <0.05 was considered statistically significant. All statistical analyses were performed using R software (Vienna, Austria; version 4.4.3).

## Results

### Baseline characteristics

After screening, 3948 oocyte retrieval cycles were included in the final analysis. The cohort was further stratified by outcome: 3672 cycles (with ≥3 oocytes retrieved) were allocated to cleavage-stage analysis and, of these, 2771 cycles (with ≥3 blastocysts cultured) and 1063 cycles were allocated to blastocyst-stage analysis and PGT-A analysis, respectively. Following exclusions for failed biopsies or unstable testing platforms, 3574 blastocysts remained for chromosomal assessment (Fig. 1).

**Figure 1.**
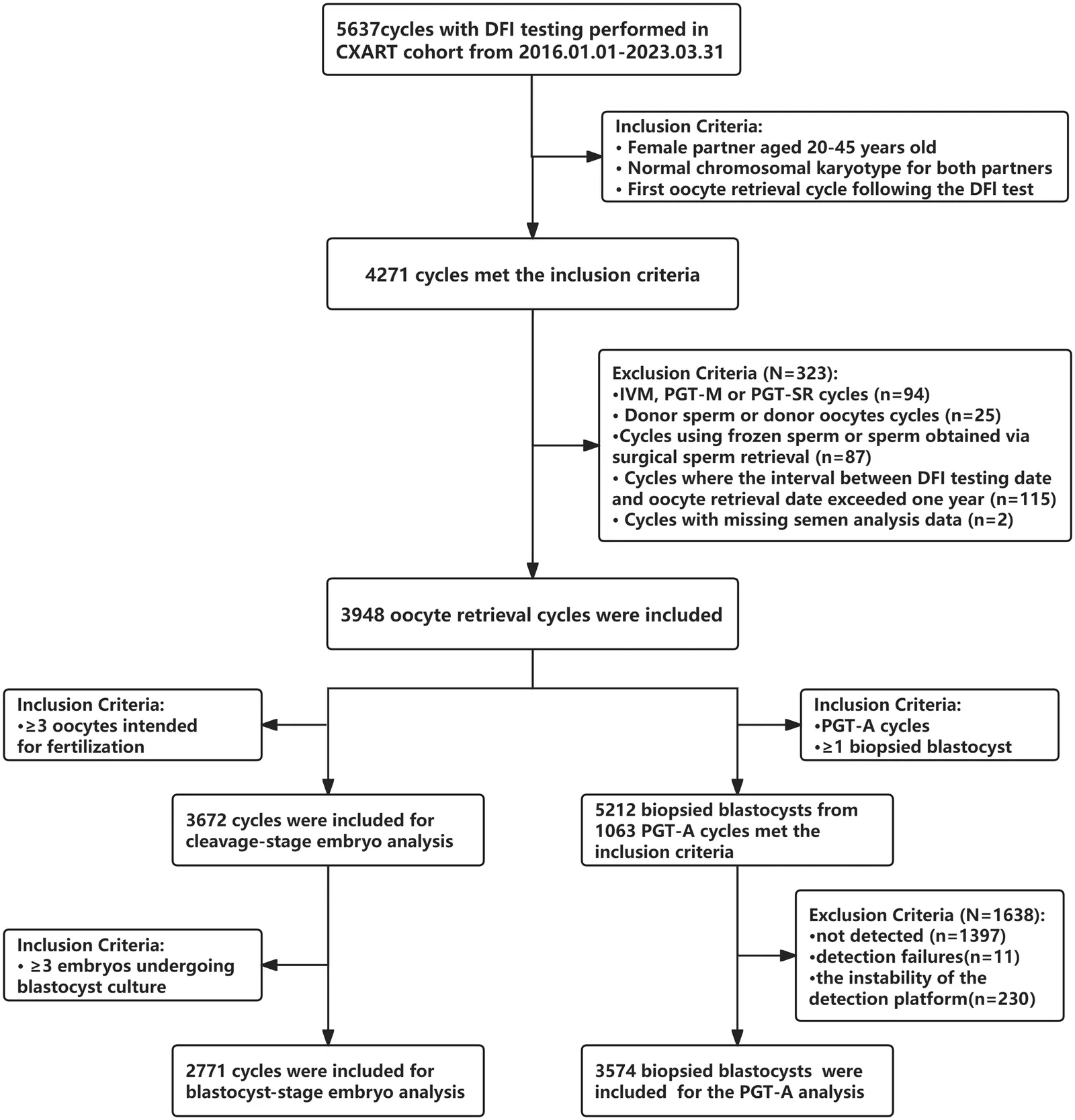
**Study flowchart**. This figure presents the flow of participants through the study, detailing inclusion and exclusion criteria. DFI, DNA fragmentation index; IVM, *in vitro* maturation; PGT-M, preimplantation genetic testing for monogenic diseases; PGT-SR, preimplantation genetic testing for structural rearrangements; PGT-A, preimplantation genetic testing for aneuploidy; CXART, CITIC-Xiangya Assisted Reproductive Technology.

Based on established clinical thresholds, these 3948 cycles were categorized into three DFI groups: low DFI (<15%, n = 2471), medium DFI (15–30%, n = 1091), and high DFI (≥30%, n = 386). Significant intergroup differences were observed in both female and male parameters (Table 1). Females in the high DFI group were significantly older (median 35.16 vs 33.00 vs 32.78 years; *P *< 0.001) and exhibited variations in BMI (*P *= 0.017), smoking rates (*P *= 0.012), anti-Müllerian hormone (AMH) levels (*P *= 0.002), and PMOS prevalence (*P *< 0.001). Male partners in the high DFI group were also older (median 38.03 vs 35.16 vs 34.12 years; *P *< 0.001) and demonstrated significantly compromised semen quality, including lower sperm concentration, total motility, and progressive motility (all *P *< 0.001, Supplementary Fig. S1). Consequently, the prevalence of SOAT increased significantly with DFI levels (8.46% vs 18.33% vs 46.89%; *P *< 0.001), leading to significant differences in the utilization of IVF versus ICSI across groups (*P < *0.001).

**Table 1.** Baseline characteristics of cycles grouped by clinical threshold of sperm DNA fragmentation index.

Characteristic	DFI < 15% (N = 2471)	15% ≤ DFI < 30% (N = 1091)	DFI ≥ 30% (N = 386)	*P* value
Female age, years	32.78 (30.14, 35.82)	33.00 (30.29, 36.67)	35.16 (31.85, 39.16)	<0.001***
Female BMI, kg/m^2^	21.63 (19.98, 23.23)	21.57 (19.96, 23.07)	21.91 (20.28, 23.44)	0.017*
Female smoking status				0.012*
No	2422 (98.02%)	1084 (99.36%)	379 (98.19%)	
Yes	49 (1.98%)	7 (0.64%)	7 (1.81%)	
Female AMH, ng/ml^a^	3.25 (1.70, 5.76)	3.18 (1.73, 5.52)	2.73 (1.41, 4.85)	0.002**
Female PMOS				<0.001***
No	2053 (83.08%)	947 (86.80%)	345 (89.38%)	
Yes	418 (16.92%)	144 (13.20%)	41 (10.62%)	
Male age, years	34.12 (31.41, 37.68)	35.16 (31.88, 39.70)	38.03 (33.87, 44.50)	<0.001***
Male BMI, kg/m^2^	24.61 (22.49, 27.04)	24.56 (22.31, 26.89)	24.68 (22.65, 26.45)	0.278
Male smoking status				<0.001***
No	1485 (60.10%)	698 (63.98%)	280 (72.54%)	
Yes	986 (39.90%)	393 (36.02%)	106 (27.46%)	
Sperm concentration, million/ml	65.30 (39.22, 102.41)	53.22 (26.62, 91.35)	31.84 (13.46, 69.98)	<0.001***
Percentage of sperm motility, %	57.23 (44.15, 69.45)	43.62 (29.78, 54.72)	25.03 (12.12, 41.75)	<0.001***
Percentage of sperm progressive motility, %	44.07 (33.17, 55.66)	33.33 (21.91, 42.65)	17.76 (6.58, 31.48)	<0.001***
Percentage of sperm with normal morphology, %	3.30 (2.80, 4.23)	3.20 (2.40, 4.20)	2.80 (2.23, 3.35)	<0.001***
Severe oligoasthenoteratospermia^b^				<0.001***
No	2262 (91.54%)	891 (81.67%)	205 (53.11%)	
Yes	209 (8.46%)	200 (18.33%)	181 (46.89%)	
Fertilization method				<0.001***
IVF	1119 (45.29%)	416 (38.13%)	40 (10.36%)	
ICSI	1097 (44.39%)	525 (48.12%)	290 (75.13%)	
IVF + ICSI	255 (10.32%)	150 (13.75%)	56 (14.51%)	

Continuous variables are presented as median (Q1, Q3), categorical variables are presented as number (percentage).

a A small amount of data was missing for AMH, accounting for 0.35% (14/3948) of the total.

b Severe oligoasthenoteratospermia was defined as a sperm concentration of <5 million/ml, or a progressive motility rate of <10%, or a normal sperm morphology rate of <2%.

AMH, anti-Müllerian hormone; DFI, DNA fragmentation index; PMOS, polyendocrine metabolic ovarian syndrome.

* *P *< 0.05.

** *P *< 0.01.

*** *P *< 0.001.

### Impact of DFI on early embryological outcomes

After multivariable adjustment, no substantial differences were observed across DFI categories in cleavage-stage parameters, including fertilization rate, normal fertilization rate, cleavage rate, or high-quality embryo rate (all *P *> 0.05; Table 2; Supplementary Fig. S2), although a significant trend toward lower fertilization rates with increasing DFI was detected (*P* for trend = 0.041). These null associations remained consistent in sensitivity analyses using propensity score OW and after restricting the cohort to cycles with DFI testing performed within 6 months prior to oocyte retrieval (Supplementary Table S1) and were not modified by female age (<35 vs ≥35 years) or male age (<40 vs ≥40 years) in prespecified subgroup analyses (Supplementary Tables S2 and S3). However, a significant interaction was observed between DFI and fertilization method on high DFI on high-quality embryo rate (*P* for interaction = 0.040; Supplementary Table S4). Notably, among men without SOAT, moderate DFI levels were associated with a significantly reduced fertilization rate compared to low DFI (adjusted odds ratio [aOR] = 0.90, 95% CI: 0.82–0.99, *P *= 0.027), and the inverse trend across DFI categories remained significant (*P* for trend = 0.040; Supplementary Table S5).

**Table 2.** Comparative analysis of embryo development parameters across sperm DNA fragmentation index groups categorized by clinical threshold.

Outcomes	Number	Rate	Adjusted OR (95% CI)	*P* value	*P* for trend
**Cleavage-stage**					
Fertilization rate					0.041*
DFI < 15%	2322	0.89 (0.78, 1.00)	Ref		
15%≤DFI < 30%	1007	0.89 (0.75, 1.00)	0.93 (0.85, 1.01)	0.080	
DFI ≥ 30%	343	0.91 (0.78, 1.00)	0.89 (0.77, 1.04)	0.137	
Normal fertilization rate					0.331
DFI < 15%	2322	0.73 (0.58, 0.86)	Ref		
15%≤DFI < 30%	1007	0.73 (0.57, 0.86)	0.96 (0.89, 1.03)	0.216	
DFI ≥ 30%	343	0.78 (0.63, 0.92)	0.98 (0.87, 1.10)	0.710	
Cleavage rate					0.755
DFI < 15%	2309	1.00 (1.00, 1.00)	Ref		
15%≤DFI < 30%	1004	1.00 (1.00, 1.00)	1.02 (0.85, 1.23)	0.798	
DFI ≥ 30%	340	1.00 (1.00, 1.00)	0.90 (0.65, 1.24)	0.505	
High-quality embryo rate					0.987
DFI < 15%	2299	0.50 (0.25, 0.75)	Ref		
15%≤DFI < 30%	996	0.50 (0.25, 0.75)	0.96 (0.88, 1.06)	0.418	
DFI ≥ 30%	338	0.54 (0.25, 0.75)	1.05 (0.90, 1.23)	0.536	
**Blastocyst-stage**					
Blastocyst formation rate					0.185
DFI < 15%	1806	0.50 (0.31, 0.67)	Ref		
15%≤DFI < 30%	725	0.46 (0.29, 0.67)	0.94 (0.87, 1.03)	0.175	
DFI ≥ 30%	240	0.50 (0.33, 0.67)	0.94 (0.82, 1.09)	0.420	
High-quality blastocyst formation rate					0.151
DFI < 15%	1806	0.13 (0.00, 0.33)	Ref		
15%≤DFI < 30%	725	0.13 (0.00, 0.30)	0.93 (0.83, 1.04)	0.189	
DFI ≥ 30%	240	0.17 (0.00, 0.33)	0.91 (0.76, 1.09)	0.308	

Continuous rates are presented as median (Q1, Q3). Each endpoint across DFI groups was analyzed using either a beta-binomial (BB) or a zero-inflated beta-binomial (ZIBB) regression model, with the final model choice determined by the better fit according to the Akaike Information Criterion (AIC). For the Cleavage-Stage outcomes (fertilization rate, normal fertilization rate, cleavage rate, and high-quality embryo rate), the multivariate models were adjusted for the following covariates: male age, male smoking status, female age, female BMI, female smoking status, polyendocrine metabolic ovarian syndrome, severe oligoasthenoteratozoospermia (SOAT), and the method of fertilization. For the Blastocyst-Stage outcomes (blastocyst formation rate and high-quality blastocyst formation rate), the multivariate models were adjusted for male age, male smoking status, female age, female BMI, SOAT, and the method of fertilization.

DFI, DNA fragmentation index; OR, odds ratio.

* *P *< 0.05.

Regarding blastocyst development (n = 2771 cycles with ≥3 embryos cultured), neither blastocyst formation rate nor high-quality blastocyst rate differed significantly across DFI groups in the overall cohort (Table 2; Supplementary Fig. S2). These findings were robust to both sensitivity analyses (Supplementary Table S1) and consistent across subgroups stratified by female age (Supplementary Table S2), fertilization method (Supplementary Table S4), and SOAT status (Supplementary Table S5). Nevertheless, in men aged <40 years, high DFI was associated with a significantly lower likelihood of forming high-quality blastocysts compared with low DFI (aOR = 0.79, 95% CI: 0.64–0.99, *P *= 0.044), accompanied by a significant inverse dose–response trend across DFI categories (*P* for trend = 0.037; Supplementary Table S3).

### Impact of DFI on PGT-A outcomes

Of 5212 embryos derived from 1063 PGT-A cycles, 3574 blastocysts were included in the final chromosomal analysis. The overall chromosomal abnormality rate was 50.31% (1798/3574), including aneuploidy (26.16%, 935/3574), mosaicism (26.50%, 947/3574), and segmental abnormalities (10.00%, 357/3574). Although the crude prevalence of chromosomal abnormalities appeared numerically higher in the high DFI group (58.24% vs 50.89% and 48.83% in the moderate and low DFI groups, respectively), this trend lacked statistical significance after multivariable adjustment (*P* for trend = 0.510). With respect to aneuploidy, the crude prevalence increased in a stepwise manner across DFI strata (24.42, 25.78, and 38.19% for low, moderate, and high DFI, respectively); however, this numerical trend did not reach statistical significance (*P* for trend = 0.222). Nevertheless, adjusted mixed-effects models, accounting for the clustering of embryos within cycles, demonstrated that high DFI independently elevated the risk of aneuploidy compared to low DFI (aOR = 1.35, 95% CI: 1.01–1.81, *P *= 0.042), whereas moderate DFI showed no substantial effect (aOR = 0.93, 95% CI: 0.76–1.13, *P *= 0.465). In contrast, no substantial associations were observed between DFI and the rates of mosaicism (26.67% vs 27.89% vs 21.98%) or segmental abnormalities (9.65% vs 10.56% vs 10.71%; all *P *> 0.05; Table 3; Fig. 2).

**Figure 2.**
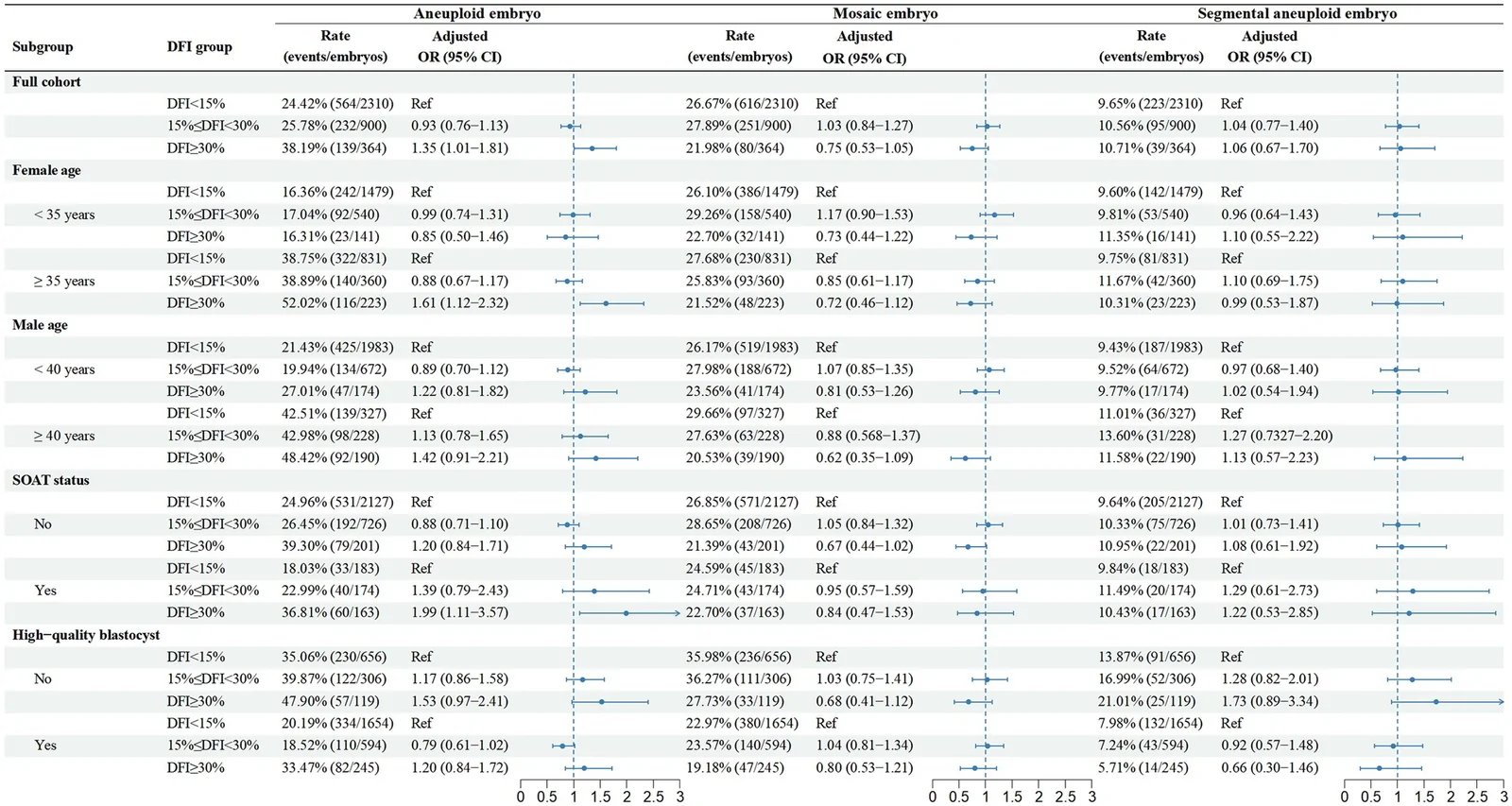
**Forest plot of subgroup analyses for the association between sperm DNA fragmentation index and embryonic chromosome integrity**. Data are presented as adjusted odds ratios (aOR) with 95% CI. Error bars represent 95% CIs. All models accounted for the non-independence of embryos within the same oocyte retrieval cycle using generalized linear mixed models. For analyses stratified by female/male age, models were adjusted for male age, male smoking status, female age, female BMI, SOAT, and blastocyst quality. For analyses stratified by SOAT status or blastocyst quality, the corresponding stratification variable was omitted from the adjustment set. aOR, adjusted odds ratio; DFI, DNA fragmentation index; SOAT, severe oligoasthenoteratozoospermia.

**Table 3.** Comparative analysis of blastocyst PGT-A results across sperm DNA fragmentation index groups categorized by clinical threshold.

Outcomes	Rate (N = 3574)	Adjusted OR (95% CI)	*P* value	*P* for trend
Whole chromosome aneuploid embryo				0.510
DFI < 15%	48.83% (1128/2310)	Ref		
15%≤DFI < 30%	50.89% (458/900)	1.00 (0.83–1.19)	0.958	
DFI ≥ 30%	58.24% (212/364)	1.14 (0.86–1.53)	0.359	
Aneuploid embryo				0.222
DFI < 15%	24.42% (564/2310)	Ref		
15%≤DFI < 30%	25.78% (232/900)	0.93 (0.76–1.13)	0.465	
DFI ≥ 30%	38.19% (139/364)	1.35 (1.01–1.81)	0.042*	
Mosaic embryo				0.267
DFI < 15%	26.67% (616/2310)	Ref		
15%≤DFI < 30%	27.89% (251/900)	1.03 (0.84–1.27)	0.757	
DFI ≥ 30%	21.98% (80/364)	0.75 (0.53–1.05)	0.089	
Segmental aneuploid embryo				0.757
DFI < 15%	9.65% (223/2310)	Ref		
15%≤DFI < 30%	10.56% (95/900)	1.04 (0.77–1.40)	0.812	
DFI ≥ 30%	10.71% (39/364)	1.06 (0.67–1.70)	0.796	

An embryo could have multiple types of chromosomal abnormalities simultaneously. Preimplantation genetic testing for aneuploidies (PGT-A) outcomes across sperm DFI groups were analyzed using generalized linear mixed models to account for the random effect of potentially submitting two or more embryos from a single cycle. The multivariate model was adjusted for male age, male smoking status, female age, female BMI, severe oligoasthenoteratozoospermia, and whether the blastocyst was of high quality.

DFI, DNA fragmentation index; OR, odds ratio.

* *P *< 0.05.

Sensitivity analyses were conducted to assess the robustness of these findings. When applying propensity score OW, the effect estimate for high DFI on aneuploidy risk was attenuated and the confidence interval widened (aOR = 1.39, 95% CI: 0.81–2.37, *P *= 0.228). This attenuation, driven by reduced effective sample size and weighting uncertainty, did not alter the direction of the association. After restricting the cohort to cycles in which DFI testing was performed within 6 months prior to oocyte retrieval, the association between high DFI and aneuploidy remained robust (aOR = 1.37, 95% CI: 1.01–1.85, *P *= 0.041; Supplementary Table S6).

Subgroup analyses revealed distinct patterns of association between DFI and embryonic aneuploidy (Fig. 2, Supplementary Tables S7, S8, S9, and S10). Compared with low DFI, high DFI was associated with a significantly increased risk of embryonic aneuploidy among women aged ≥35 years (52.02% vs 38.75%; aOR = 1.61, 95% CI: 1.12–2.32, *P *= 0.010; Supplementary Table S7). A similar elevation in aneuploidy risk was observed among men with SOAT (36.81% vs 18.03%; aOR = 1.99, 95% CI: 1.11–3.57, *P *= 0.022; Supplementary Table S9), in whom a significant dose–response trend was also detected across DFI categories (*P* for trend = 0.022).

A significant interaction was observed between DFI and male age regarding whole-chromosome aneuploidy (*P* for interaction = 0.005). Although the association was not substantial within either age stratum, point estimates suggested a differential effect: high DFI was associated with a non-substantial reduction in whole-chromosome aneuploidy risk among men aged <40 years (aOR < 1), whereas it pointed to an increased risk among men aged ≥40 years (aOR > 1; Supplementary Table S8). Similarly, a significant interaction emerged between DFI and blastocyst quality regarding segmental aneuploidy (*P* for interaction <0.001). Point estimates indicated that high DFI was associated with a non-substantial reduction in segmental aneuploidy among high-quality blastocysts (aOR < 1) and a non-substantial increase among low-quality blastocysts (aOR > 1; Supplementary Table S10).

## Discussion

Drawing on a large cohort of 3948 couples, this study demonstrates that sperm DFI is an independent risk factor for embryonic aneuploidy (aOR = 1.35). Crucially, this risk exhibits a synergistic amplification in specific contexts: when the female partner is aged ≥35 years (aOR = 1.61) or when the male partner presents with SOAT (aOR = 1.99). These findings contribute large-scale, real-world evidence to the ongoing debate on the link between DFI and embryonic chromosomal integrity.

The underlying mechanisms are likely multifaceted. First, high DFI may serve as a biomarker of intrinsic defects in spermatogenesis, such as meiotic errors or dysregulated apoptosis, often accompanied by elevated oxidative stress in semen (Gharagozloo and Aitken, 2011; Bisht *et al.*, 2017). These processes may inherently increase the production of gametes with numerical chromosomal abnormalities. Second, Reactive oxygen species (ROS) originating from sperm may disrupt the microtubule stability of the zygotic mitotic spindle, directly interfering with accurate chromosome segregation and thereby increasing aneuploidy risk (Simões *et al.*, 2013). However, the most critical factor is the repair capacity of the oocyte and its threshold effect. Sperm DNA damage repair (DDR) is entirely dependent on the oocyte, and in women of advanced age, the DDR capacity of oocytes significantly declines due to down-regulation of related genes (Horta *et al.*, 2020). Unrepaired damage exceeding a critical threshold adversely affects embryo development (Marinaro, 2023). In this study, when advanced maternal age coexisted with high paternal DFI, the risk of embryonic aneuploidy increased sharply. This is a direct manifestation of repair system overload when an oocyte with declining repair capacity encounters sperm with a high damage load (Setti *et al.*, 2021; Fu *et al.*, 2024). Furthermore, the aneuploidy risk was further exacerbated in men with SOAT combined with high DFI, suggesting that severe spermatogenic dysfunction and high DFI may share a deeper pathological basis on embryonic chromosomal stability.

We acknowledge that sperm preparation (density gradient centrifugation or swim-up) can lower the DFI of the fraction ultimately used for ICSI relative to the diagnostic raw-semen value. However, importantly, the relative ranking of DFI among patients is largely preserved after selection: those with high baseline DFI retain higher post-processing DFI than those with low baseline DFI (Muratori *et al.*, 2019). Raw-semen DFI therefore remains a clinically meaningful biomarker, as it reflects the constitutive genotoxic burden arising from defective chromatin remodeling and oxidative stress during spermiogenesis and epididymal transit (Tomlinson *et al.*, 2001), and it is the parameter most commonly measured before treatment in routine practice. Consistent with previous reports, elevated DFI was associated with poorer conventional semen parameters (Liu *et al.*, 2023; Yang *et al.*, 2024), further underscoring its role as a marker of impaired spermatogenesis.

We also observed a negative impact of high DFI on high-quality blastocyst formation, in line with earlier findings (Wang *et al.*, 2022), supporting the notion that sperm DNA integrity is fundamental to later-stage embryonic development. Notably, early cleavage processes were not significantly affected, which may be explained by an ‘oocyte-mediated repair buffer’ effect. The oocyte cytoplasm contains a comprehensive DDR system, and all DNA repair pathways are of maternal origin during fertilization and prior to embryonic genome activation (González-Marín *et al.*, 2012; Martin *et al.*, 2019). This system can effectively repair mild-to-moderate paternal DNA damage shortly after fertilization, maintaining normal cleavage progression at the morphological level. In contrast, the blastocyst formation depends on precise paternal genome activation and complex epigenetic reprogramming, making it highly sensitive to genomic integrity. When sperm DNA damage exceeds the oocyte’s repair capacity, it may interfere with paternal chromatin function, ultimately manifesting as reduced blastocyst quality. This suggests that the negative effects of DFI primarily emerge after zygotic genome activation.

Given this background, we next sought to clarify the mechanisms underlying our finding that high DFI was associated specifically with aneuploidy, rather than with segmental or mosaic abnormalities. First, our finding that high DFI is associated with an increased rate of whole-chromosome aneuploidy is consistent with the majority of published reports (Gao *et al.*, 2023; Wan *et al.*, 2025). This association has been robustly demonstrated across multiple cohorts, including in oocyte donation cycles where maternal age is excluded as a confounding factor (García-Ferreyra *et al.*, 2015). Second, we acknowledge that some studies have also reported an association between DFI and segmental aneuploidy (Gao *et al.*, 2023). We attribute the discrepancy between our findings and these reports primarily to a methodological difference: in the present study, only the four highest-grade blastocysts were selected for PGT-A, and segmental and mosaic abnormalities are known to be considerably less frequent in morphologically and/or chromosomally selected embryos (Gao *et al*., 2023). Importantly, despite this low background rate, whole-chromosome aneuploidy was still significantly elevated in the high-DFI group.

These findings may inform ART practice by identifying subgroups who could benefit from more intensive counseling and risk stratification. DFI assessment may be a useful adjunct to conventional semen analysis, particularly for couples with advanced maternal age or severe male factor infertility. Given that female age remains the primary determinant of embryonic ploidy (Kong *et al.*, 2026), identifying high-risk couples allows for informed counseling regarding the potential utility of PGT-A. Then, for patients with elevated DFI, preconception interventions, such as lifestyle modifications, antioxidant therapy, or advanced sperm selection techniques, warrant further investigation as potential strategies to improve sperm genomic integrity, though their clinical efficacy requires confirmation in prospective studies.

This study has several strengths. First, it leverages a large-scale, real-world cohort of 3948 ART cycles and 3574 biopsied blastocysts, providing robust statistical power to detect clinically relevant associations. Second, we employed advanced statistical modeling, specifically GLMMs, to account for the clustering of embryos within cycles and the overdispersion of proportional data. Third, the integration of PGT-A data allowed for a precise assessment of the impact of DFI on specific chromosomal abnormality subtypes. However, several limitations warrant acknowledgment. As a single-center retrospective study, residual confounding cannot be entirely excluded, limiting causal inference. Furthermore, in patients of severe SOAT, limited sperm availability often restricted comprehensive DFI testing, which may constrain the generalizability of our findings to extreme male factor infertility. Moreover, while we identified a substantial association between DFI and aneuploidy, the lack of molecular assessments, such as sperm epigenetic modifications or oocyte-specific DNA repair capacity, limits our ability to mechanistically explain the observed synergistic effect with advanced maternal age.

## Conclusions

In summary, this large-scale analysis demonstrates that elevated sperm DFI is an independent risk factor for impaired high-quality blastocyst formation and increased embryonic aneuploidy. These risks are significantly amplified in subgroups characterized by advanced maternal age or SOAT. Given the observational design, causal inference cannot be established, and residual confounding cannot be excluded. Whether routine DFI testing improves ART outcomes, and whether risk stratification based on DFI should guide specific interventions such as PGT-A utilization, pre-cycle management, or modified sperm selection techniques remains a hypothesis that requires validation through well-designed prospective cohorts and randomized controlled trials. Future well-designed prospective cohorts and randomized controlled trials are essential to establish causal pathways and cost-effectiveness of DFI-guided treatment strategies before widespread implementation.

## Supplementary Material

hoag082_Supplementary_Data

## Data Availability

The data used in this retrospective study originate from the CITIC-Xiangya Assisted Reproductive Technology (CXART) cohort (ClinicalTrials.gov identifier: NCT05404464). Due to patient confidentiality requirements and ethical restrictions, the raw clinical datasets are not publicly available. De-identified data relevant to this study can be made available upon reasonable request from the corresponding author, pending review and formal approval by the Ethics Committee of the Reproductive and Genetic Hospital of CITIC-Xiangya.
