# Supplementary material for: High sperm DNA fragmentation is associated with increased embryonic chromosomal abnormality: a retrospective study of 8 years involving 5637 couples undergoing ART
Source: Hum Reprod Open. 2026 Sep 16;2026(4):hoag082. doi: 10.1093/hropen/hoag082 (PMC13658023; doi:10.1093/hropen/hoag082)
Supplement: Supplementary file 1 [file hoag082_supplementary_data.zip › Supplementary_Tables_EO.docx]

| **Supplementary Table S1.** Sensitivity analysis of Embryo Development Parameters Across Sperm DNA Fragmentation Index Groups Categorized by Clinical Threshold. | | | | |
| --- | --- | --- | --- | --- |
| **Outcomes** | **Propensity Score Overlap Weighting** | | **DFI testing and oocyte retrieval interval limited to ≤ 6 months*** | |
|  | **Adjusted OR (95% CI)** | ***P* value** | **Adjusted OR (95% CI)** | ***P* value** |
| **Cleavage-Stage** |  |  |  |  |
| Fertilization rate |  |  |  |  |
| DFI<15% | Ref |  | Ref |  |
| 15%≤DFI<30% | 0.93 (0.72, 1.21) | 0.602 | 0.93 (0.85, 1.02) | 0.118 |
| DFI≥30% | 0.91 (0.70, 1.18) | 0.476 | 0.91 (0.78, 1.07) | 0.238 |
| Normal fertilization rate |  |  |  |  |
| DFI<15% | Ref |  | Ref |  |
| 15%≤DFI<30% | 0.97 (0.80, 1.19) | 0.801 | 0.97 (0.90, 1.04) | 0.384 |
| DFI≥30% | 1.00 (0.82, 1.22) | 0.991 | 1.01 (0.89, 1.15) | 0.854 |
| Cleavage rate |  |  |  |  |
| DFI<15% | Ref |  | Ref |  |
| 15%≤DFI<30% | 0.95 (0.55, 1.64) | 0.863 | 1.00 (0.82, 1.21) | 0.975 |
| DFI≥30% | 0.85 (0.50, 1.46) | 0.563 | 0.89 (0.64, 1.24) | 0.490 |
| High-quality embryo rate |  |  |  |  |
| DFI<15% | Ref |  | Ref |  |
| 15%≤DFI<30% | 1.03 (0.79, 1.34) | 0.809 | 0.97 (0.88, 1.07) | 0.497 |
| DFI≥30% | 1.06 (0.81, 1.38) | 0.668 | 1.02 (0.87, 1.20) | 0.778 |
| **Blastocyst-Stage** |  |  |  |  |
| Blastocyst formation rate |  |  |  |  |
| DFI<15% | Ref |  | Ref |  |
| 15%≤DFI<30% | 0.99 (0.78, 1.25) | 0.904 | 0.95 (0.87, 1.03) | 0.214 |
| DFI≥30% | 0.96 (0.75, 1.22) | 0.709 | 0.93 (0.80, 1.07) | 0.301 |
| High-quality blastocyst formation rate |  |  |  |  |
| DFI<15% | Ref |  | Ref |  |
| 15%≤DFI<30% | 0.97 (0.71, 1.33) | 0.869 | 0.94 (0.84, 1.06) | 0.312 |
| DFI≥30% | 0.92 (0.68, 1.26) | 0.624 | 0.89 (0.74, 1.08) | 0.248 |

*, sperm DNA fragmentation index (DFI) testing was restricted to within six months before oocyte retrieval. A total of 3365 cycles were available for cleavage-stage outcome analyses, and 2532 cycles for blastocyst-stage outcome analyses.

Each endpoint across sperm DFI groups was analyzed using either a beta-binomial (BB) or a zero-inflated beta-binomial (ZIBB) regression model, with the final model choice determined by the better fit according to the Akaike Information Criterion (AIC). For the Cleavage-Stage outcomes (fertilization rate, normal fertilization rate, cleavage rate, and high-quality embryo rate), covariates adjusted for in both propensity score and multivariable analyses included male age, male smoking status, female age, female body mass index, female smoking status, polyendocrine metabolic ovarian syndrome, severe oligoasthenoteratozoospermia, and the method of fertilization. For the Blastocyst-Stage outcomes (blastocyst formation rate and high-quality blastocyst formation rate), covariates adjusted for in both propensity score and multivariable analyses included male age, male smoking status, female age, female body mass index, severe oligoasthenoteratozoospermia, and the method of fertilization. CI, confidence interval; DFI, DNA fragmentation index; OR, odds ratio.

| **Supplementary Table S2.** Comparative Analysis of Embryo Development Parameters Across Sperm DNA Fragmentation Index Groups Stratified by Female Age | | | | | | | | | | | | |
| --- | --- | --- | --- | --- | --- | --- | --- | --- | --- | --- | --- | --- |
| **Outcomes** | **Female age-stratified subgroups** | | | | | | | | | | | |
|  | **Female age <35** | | | | | **Female age ≥35** | | | | | | ***P* for interaction** |
|  | **Number** | **Rate** | **Adjusted OR (95% CI)** | ***P* value** | ***P* for trend** | **Number** | **Rate** | **Adjusted OR (95% CI)** | ***P* value** | ***P* for trend** | |  |
| **Cleavage-Stage** |  |  |  |  |  |  |  |  |  | |  |  |
| Fertilization |  |  |  |  | 0.153 |  |  |  |  | | 0.103 | 0.822 |
| DFI<15% | 1673 | 0.89 (0.77, 1.00) | Ref |  |  | 649 | 0.91 (0.80, 1.00) | Ref |  | |  |  |
| 15%≤DFI<30% | 669 | 0.88 (0.75, 1.00) | 0.91 (0.82, 1.01) | 0.066 |  | 338 | 0.92 (0.80, 1.00) | 0.97 (0.82, 1.15) | 0.705 | |  |  |
| DFI≥30% | 187 | 0.90 (0.80, 1.00) | 0.96 (0.79, 1.17) | 0.671 |  | 156 | 0.91 (0.75, 1.00) | 0.79 (0.62, 1.01) | 0.058 | |  |  |
| Normal fertilization |  |  |  |  | 0.361 |  |  |  |  | | 0.640 | 0.891 |
| DFI<15% | 1673 | 0.71 (0.57, 0.85) | Ref |  |  | 649 | 0.75 (0.60, 0.89) | Ref |  | |  |  |
| 15%≤DFI<30% | 669 | 0.71 (0.56, 0.86) | 0.95 (0.87, 1.03) | 0.191 |  | 338 | 0.75 (0.60, 0.90) | 0.98 (0.86, 1.11) | 0.744 | |  |  |
| DFI≥30% | 187 | 0.78 (0.63, 0.92) | 0.99 (0.84, 1.16) | 0.890 |  | 156 | 0.78 (0.63, 0.92) | 0.96 (0.79, 1.16) | 0.678 | |  |  |
| Cleavage |  |  |  |  | 0.906 |  |  |  |  | | 0.605 | 0.633 |
| DFI<15% | 1664 | 1.00 (1.00, 1.00) | Ref |  |  | 645 | 1.00 (1.00, 1.00) | Ref |  | |  |  |
| 15%≤DFI<30% | 669 | 1.00 (1.00, 1.00) | 1.01 (0.82, 1.26) | 0.904 |  | 337 | 1.00 (1.00, 1.00) | 1.04 (0.72, 1.51) | 0.843 | |  |  |
| DFI≥30% | 187 | 1.00 (1.00, 1.00) | 0.94 (0.63, 1.41) | 0.770 |  | 153 | 1.00 (1.00, 1.00) | 0.80 (0.47, 1.37) | 0.425 | |  |  |
| High-quality embryo |  |  |  |  | 0.730 |  |  |  |  | |  | 0.587 |
| DFI<15% | 1660 | 0.50 (0.25, 0.75) | Ref |  |  | 639 | 0.50 (0.25, 0.76) | Ref |  | | 0.609 |  |
| 15%≤DFI<30% | 661 | 0.50 (0.24, 0.75) | 0.96 (0.86, 1.08) | 0.495 |  | 335 | 0.50 (0.25, 0.75) | 0.97 (0.81, 1.15) | 0.697 | |  |  |
| DFI≥30% | 186 | 0.50 (0.20, 0.75) | 1.01 (0.82, 1.24) | 0.923 |  | 152 | 0.57 (0.30, 0.75) | 1.11 (0.87, 1.41) | 0.408 | |  |  |
| **Blastocyst-Stage** |  |  |  |  |  |  |  |  |  | |  |  |
| Blastocyst formation |  |  |  |  | 0.155 |  |  |  |  | | 0.935 | 0.602 |
| DFI<15% | 1287 | 0.50 (0.31, 0.67) | Ref |  |  | 519 | 0.50 (0.30, 0.67) | Ref |  | |  |  |
| 15%≤DFI<30% | 489 | 0.50 (0.30, 0.67) | 0.97 (0.88, 1.07) | 0.541 |  | 236 | 0.40 (0.27, 0.67) | 0.90 (0.78, 1.05) | 0.188 | |  |  |
| DFI≥30% | 131 | 0.50 (0.27, 0.67) | 0.86 (0.72, 1.04) | 0.121 |  | 109 | 0.50 (0.33, 0.67) | 1.09 (0.88, 1.35) | 0.424 | |  |  |
| High-quality blastocyst formation |  |  |  |  | 0.069 |  |  |  |  | | 0.498 | 0.841 |
| DFI<15% | 1287 | 0.13 (0.00, 0.33) | Ref |  |  | 519 | 0.14 (0.00, 0.33) | Ref |  | |  |  |
| 15%≤DFI<30% | 489 | 0.13 (0.00, 0.31) | 0.94 (0.82, 1.07) | 0.333 |  | 236 | 0.13 (0.00, 0.30) | 0.94 (0.77, 1.15) | 0.572 | |  |  |
| DFI≥30% | 131 | 0.13 (0.00, 0.33) | 0.80 (0.63, 1.02) | 0.072 |  | 109 | 0.18 (0.00, 0.33) | 1.16 (0.89, 1.53) | 0.270 | |  |  |

Continuous rates are presented as median (Q1, Q3). Each endpoint across sperm DFI groups was analyzed using either a beta-binomial (BB) or a zero-inflated beta-binomial (ZIBB) regression model, with the final model choice determined by the better fit according to the Akaike Information Criterion (AIC). For the Cleavage-Stage outcomes (fertilization rate, normal fertilization rate, cleavage rate, and high-quality embryo rate), the multivariate models were adjusted for the following covariates: male age, male smoking status, female age, female body mass index, female smoking status, polyendocrine metabolic ovarian syndrome, severe oligoasthenoteratozoospermia, and the method of fertilization. For the Blastocyst-Stage outcomes (blastocyst formation rate and high-quality blastocyst formation rate), the multivariate models were adjusted for male age, male smoking status, female age, female body mass index, severe oligoasthenoteratozoospermia, and the method of fertilization. CI, confidence interval; DFI, DNA fragmentation index; OR, odds ratio.

| **Supplementary Table S3.** Comparative Analysis of Embryo Development Parameters Across Sperm DNA Fragmentation Index Groups Stratified by Male Age | | | | | | | | | | | |
| --- | --- | --- | --- | --- | --- | --- | --- | --- | --- | --- | --- |
| **Outcomes** | **Male age-stratified subgroups** | | | | | | | | | | |
|  | **Male age <40** | | | | | **Male age ≥40** | | | | | ***P* for interaction** |
|  | **Number** | **Rate** | **Adjusted OR (95% CI)** | ***P* value** | ***P* for trend** | **Number** | **Rate** | **Adjusted OR (95% CI)** | ***P* value** | ***P* for trend** |  |
| **Cleavage-Stage** |  |  |  |  |  |  |  |  |  |  |  |
| Fertilization |  |  |  |  | 0.116 |  |  |  |  | 0.490 | 0.435 |
| DFI<15% | 2016 | 0.89 (0.78, 1.00) | Ref |  |  | 306 | 0.91 (0.78, 1.00) | Ref |  |  |  |
| 15%≤DFI<30% | 787 | 0.88 (0.75, 1.00) | 0.92 (0.84, 1.02) | 0.101 |  | 220 | 0.91 (0.80, 1.00) | 0.92 (0.73, 1.16) | 0.492 |  |  |
| DFI≥30% | 214 | 0.90 (0.80, 1.00) | 0.93 (0.78, 1.11) | 0.413 |  | 129 | 0.92 (0.75, 1.00) | 0.92 (0.68, 1.23) | 0.569 |  |  |
| Normal fertilization |  |  |  |  | 0.353 |  |  |  |  | 0.236 | 0.182 |
| DFI<15% | 2016 | 0.73 (0.58, 0.86) | Ref |  |  | 306 | 0.71 (0.59, 0.88) | Ref |  |  |  |
| 15%≤DFI<30% | 787 | 0.73 (0.58, 0.86) | 0.96 (0.89, 1.03) | 0.240 |  | 220 | 0.75 (0.57, 0.88) | 1.00 (0.84, 1.19) | 0.965 |  |  |
| DFI≥30% | 214 | 0.78 (0.64, 0.90) | 0.98 (0.85, 1.13) | 0.776 |  | 129 | 0.80 (0.60, 0.95) | 1.18 (0.94, 1.49) | 0.157 |  |  |
| Cleavage |  |  |  |  | 0.565 |  |  |  |  | 0.580 | 0.281 |
| DFI<15% | 2006 | 1.00 (1.00, 1.00) | Ref |  |  | 303 | 1.00 (1.00, 1.00) | Ref |  |  |  |
| 15%≤DFI<30% | 786 | 1.00 (1.00, 1.00) | 0.96 (0.79, 1.17) | 0.670 |  | 218 | 1.00 (1.00, 1.00) | 1.50 (0.91, 2.48) | 0.111 |  |  |
| DFI≥30% | 214 | 1.00 (1.00, 1.00) | 0.92 (0.63, 1.33) | 0.645 |  | 126 | 1.00 (1.00, 1.00) | 1.00 (0.55, 1.82) | 0.998 |  |  |
| High-quality embryo |  |  |  |  | 0.553 |  |  |  |  | 0.308 | 0.462 |
| DFI<15% | 1998 | 0.50 (0.25, 0.75) | Ref |  |  | 301 | 0.50 (0.20, 0.78) | Ref |  |  |  |
| 15%≤DFI<30% | 779 | 0.50 (0.25, 0.73) | 0.94 (0.85, 1.05) | 0.275 |  | 217 | 0.50 (0.23, 0.79) | 1.04 (0.82, 1.32) | 0.728 |  |  |
| DFI≥30% | 213 | 0.50 (0.22, 0.75) | 1.01 (0.84, 1.22) | 0.924 |  | 125 | 0.56 (0.30, 0.75) | 1.17 (0.88, 1.56) | 0.288 |  |  |
| **Blastocyst-Stage** |  |  |  |  |  |  |  |  |  |  |  |
| Blastocyst formation |  |  |  |  | 0.061 |  |  |  |  | 0.450 | 0.874 |
| DFI<15% | 1,587 | 0.50 (0.31, 0.67) | Ref |  |  | 219 | 0.43 (0.29, 0.67) | Ref |  |  |  |
| 15%≤DFI<30% | 571 | 0.50 (0.29, 0.67) | 0.94 (0.86, 1.03) | 0.183 |  | 154 | 0.40 (0.29, 0.67) | 1.00 (0.82, 1.23) | 0.970 |  |  |
| DFI≥30% | 145 | 0.47 (0.32, 0.67) | 0.88 (0.74, 1.04) | 0.123 |  | 95 | 0.50 (0.33, 0.67) | 1.12 (0.86, 1.46) | 0.386 |  |  |
| High-quality blastocyst formation |  |  |  |  | 0.037* |  |  |  |  | 0.302 | 0.838 |
| DFI<15% | 1,587 | 0.13 (0.00, 0.33) | Ref |  |  | 219 | 0.10 (0.00, 0.30) | Ref |  |  |  |
| 15%≤DFI<30% | 571 | 0.13 (0.00, 0.33) | 0.93 (0.82, 1.05) | 0.258 |  | 154 | 0.09 (0.00, 0.29) | 0.98 (0.74, 1.30) | 0.907 |  |  |
| DFI≥30% | 145 | 0.14 (0.00, 0.33) | 0.79 (0.64, 0.99) | 0.044* |  | 95 | 0.18 (0.00, 0.33) | 1.23 (0.88, 1.72) | 0.229 |  |  |

Continuous rates are presented as median (Q1, Q3). Each endpoint across sperm DFI groups was analyzed using either a beta-binomial (BB) or a zero-inflated beta-binomial (ZIBB) regression model, with the final model choice determined by the better fit according to the Akaike Information Criterion (AIC). For the Cleavage-Stage outcomes (fertilization rate, normal fertilization rate, cleavage rate, and high-quality embryo rate), the multivariate models were adjusted for the following covariates: male age, male smoking status, female age, female body mass index, female smoking status,polyendocrine metabolic ovarian syndrome, severe oligoasthenoteratozoospermia, and the method of fertilization. For the Blastocyst-Stage outcomes (blastocyst formation rate and high-quality blastocyst formation rate), the multivariate models were adjusted for male age, male smoking status, female age, female body mass index, severe oligoasthenoteratozoospermia, and the method of fertilization. CI, confidence interval; DFI, DNA fragmentation index; OR, odds ratio. **P*<0.05.

| **Supplementary Table S4.** Comparative Analysis of Embryo Development Parameters Across Sperm DNA Fragmentation Index Groups Stratified by Fertilization Method | | | | | | | | | | | |
| --- | --- | --- | --- | --- | --- | --- | --- | --- | --- | --- | --- |
| **Outcomes** | **Fertilization method-stratified subgroups** | | | | | | | | | | |
|  | **IVF** | | | | | **ICSI** | | | | | ***P* for interaction** |
|  | **Number** | **Rate** | **Adjusted OR (95% CI)** | ***P* value** | ***P* for trend** | **Number** | **Rate** | **Adjusted OR (95% CI)** | ***P* value** | ***P* for trend** |  |
| **Cleavage-Stage** |  |  |  |  |  |  |  |  |  |  |  |
| Fertilization |  |  |  |  | 0.463 |  |  |  |  | 0.083 | 0.853 |
| DFI<15% | 1062 | 0.86 (0.73, 0.95) | Ref |  |  | 1007 | 0.94 (0.85, 1.00) | Ref |  |  |  |
| 15%≤DFI<30% | 392 | 0.83 (0.71, 1.00) | 0.96 (0.85, 1.09) | 0.530 |  | 468 | 0.93 (0.82, 1.00) | 0.87 (0.75, 1.00) | 0.054 |  |  |
| DFI≥30% | 34 | 0.85 (0.67, 1.00) | 0.91 (0.60, 1.38) | 0.656 |  | 255 | 0.93 (0.80, 1.00) | 0.88 (0.72, 1.08) | 0.216 |  |  |
| Normal fertilization |  |  |  |  | 0.478 |  |  |  |  | 0.606 | 0.877 |
| DFI<15% | 1062 | 0.64 (0.50, 0.75) | Ref |  |  | 1007 | 0.83 (0.70, 0.95) | Ref |  |  |  |
| 15%≤DFI<30% | 392 | 0.64 (0.50, 0.75) | 0.95 (0.86, 1.05) | 0.292 |  | 468 | 0.82 (0.70, 0.92) | 0.95 (0.85, 1.07) | 0.414 |  |  |
| DFI≥30% | 34 | 0.67 (0.55, 0.80) | 1.07 (0.77, 1.47) | 0.697 |  | 255 | 0.82 (0.67, 1.00) | 0.98 (0.83, 1.15) | 0.805 |  |  |
| Cleavage |  |  |  |  | 0.686 |  |  |  |  | 0.700 | 0.600 |
| DFI<15% | 1050 | 1.00 (1.00, 1.00) | Ref |  |  | 1006 | 1.00 (1.00, 1.00) | Ref |  |  |  |
| 15%≤DFI<30% | 391 | 1.00 (1.00, 1.00) | 1.23 (0.94, 1.62) | 0.131 |  | 466 | 1.00 (1.00, 1.00) | 0.90 (0.66, 1.23) | 0.504 |  |  |
| DFI≥30% | 33 | 1.00 (0.92, 1.00) | 0.55 (0.29, 1.05) | 0.070 |  | 253 | 1.00 (1.00, 1.00) | 0.97 (0.62, 1.51) | 0.884 |  |  |
| High-quality embryo |  |  |  |  | 0.658 |  |  |  |  | 0.345 | 0.040* |
| DFI<15% | 1043 | 0.50 (0.25, 0.78) | Ref |  |  | 1003 | 0.52 (0.29, 0.75) | Ref |  |  |  |
| 15%≤DFI<30% | 385 | 0.50 (0.22, 0.75) | 0.91 (0.78, 1.05) | 0.193 |  | 464 | 0.50 (0.25, 0.75) | 0.93 (0.81, 1.07) | 0.301 |  |  |
| DFI≥30% | 33 | 0.67 (0.33, 0.80) | 1.34 (0.84, 2.12) | 0.215 |  | 251 | 0.50 (0.25, 0.75) | 0.94 (0.78, 1.13) | 0.501 |  |  |
| **Blastocyst-Stage** |  |  |  |  |  |  |  |  |  |  |  |
| Blastocyst formation |  |  |  |  | 0.152 |  |  |  |  | 0.365 | 0.135 |
| DFI<15% | 716 | 0.43 (0.25, 0.60) | Ref |  |  | 896 | 0.56 (0.37, 0.71) | Ref |  |  |  |
| 15%≤DFI<30% | 237 | 0.40 (0.20, 0.57) | 0.87 (0.76, 1.00) | 0.057 |  | 394 | 0.50 (0.33, 0.69) | 0.95 (0.85, 1.06) | 0.336 |  |  |
| DFI≥30% | 16 | 0.42 (0.29, 0.55) | 1.14 (0.69, 1.88) | 0.612 |  | 197 | 0.50 (0.33, 0.67) | 0.95 (0.81, 1.11) | 0.524 |  |  |
| High-quality blastocyst formation |  |  |  |  | 0.568 |  |  |  |  | 0.067 | 0.198 |
| DFI<15% | 716 | 0.00 (0.00, 0.20) | Ref |  |  | 896 | 0.25 (0.00, 0.42) | Ref |  |  |  |
| 15%≤DFI<30% | 237 | 0.00 (0.00, 0.18) | 0.87 (0.69, 1.08) | 0.211 |  | 394 | 0.20 (0.00, 0.38) | 0.89 (0.77, 1.02) | 0.094 |  |  |
| DFI≥30% | 16 | 0.06 (0.00, 0.32) | 1.57 (0.75, 3.28) | 0.232 |  | 197 | 0.18 (0.00, 0.33) | 0.87 (0.71, 1.06) | 0.160 |  |  |

Continuous rates are presented as median (Q1, Q3). Each endpoint across sperm DFI groups was analyzed using either a beta-binomial (BB) or a zero-inflated beta-binomial (ZIBB) regression model, with the final model choice determined by the better fit according to the Akaike Information Criterion (AIC). For the Cleavage-Stage outcomes (fertilization rate, normal fertilization rate, cleavage rate, and high-quality embryo rate), the multivariate models were adjusted for the following covariates: male age, male smoking status, female age, female body mass index, female smoking status, polyendocrine metabolic ovarian syndrome, severe oligoasthenoteratozoospermia. For the Blastocyst-Stage outcomes (blastocyst formation rate and high-quality blastocyst formation rate), the multivariate models were adjusted for male age, male smoking status, female age, female body mass index, and severe oligoasthenoteratozoospermia. CI, confidence interval; DFI, DNA fragmentation index; ICSI, intracytoplasmic sperm injection; IVF, in vitro fertilization; OR, odds ratio. **P*<0.05.

| **Supplementary Table S5.** Comparative Analysis of Embryo Development Parameters Across Sperm DNA Fragmentation Index Groups Stratified by Severe Oligoasthenoteratozoospermia Status. | | | | | | | | | | | |
| --- | --- | --- | --- | --- | --- | --- | --- | --- | --- | --- | --- |
| **Outcomes** | **Severe oligoasthenoteratospermia status-stratified subgroups** | | | | | | | | | | |
|  | **No** | | | | | **Yes** | | | | | ***P* for interaction** |
|  | **Number** | **Rate** | **Adjusted OR (95% CI)** | ***P* value** | ***P* for trend** | **Number** | **Rate** | **Adjusted OR (95% CI)** | ***P* value** | ***P* for trend** |  |
| **Cleavage-Stage** |  |  |  |  |  |  |  |  |  |  |  |
| Fertilization |  |  |  |  | 0.040* |  |  |  |  | 0.813 | 0.528 |
| DFI<15% | 2124 | 0.89 (0.78, 1.00) | Ref |  |  | 198 | 0.89 (0.76, 1.00) | Ref |  |  |  |
| 15%≤DFI<30% | 821 | 0.89 (0.75, 1.00) | 0.90 (0.82, 0.99) | 0.027* |  | 186 | 0.91 (0.80, 1.00) | 1.11 (0.87, 1.40) | 0.413 |  |  |
| DFI≥30% | 181 | 0.91 (0.78, 1.00) | 0.92 (0.75, 1.11) | 0.378 |  | 162 | 0.92 (0.78, 1.00) | 0.94 (0.72, 1.24) | 0.682 |  |  |
| Normal fertilization |  |  |  |  | 0.213 |  |  |  |  | 0.737 | 0.469 |
| DFI<15% | 2124 | 0.72 (0.58, 0.86) | Ref |  |  | 198 | 0.75 (0.60, 0.86) | Ref |  |  |  |
| 15%≤DFI<30% | 821 | 0.71 (0.56, 0.86) | 0.94 (0.87, 1.01) | 0.085 |  | 186 | 0.79 (0.64, 0.89) | 1.09 (0.90, 1.31) | 0.369 |  |  |
| DFI≥30% | 181 | 0.75 (0.63, 0.92) | 0.99 (0.85, 1.16) | 0.912 |  | 162 | 0.81 (0.63, 0.92) | 1.02 (0.83, 1.27) | 0.831 |  |  |
| Cleavage |  |  |  |  | 0.620 |  |  |  |  | 0.705 | 0.557 |
| DFI<15% | 2,114 | 1.00 (1.00, 1.00) | Ref |  |  | 195 | 1.00 (1.00, 1.00) | Ref |  |  |  |
| 15%≤DFI<30% | 819 | 1.00 (1.00, 1.00) | 0.98 (0.81, 1.19) | 0.851 |  | 185 | 1.00 (1.00, 1.00) | 1.43 (0.82, 2.48) | 0.209 |  |  |
| DFI≥30% | 179 | 1.00 (1.00, 1.00) | 0.89 (0.60, 1.32) | 0.562 |  | 161 | 1.00 (1.00, 1.00) | 1.07 (0.59, 1.92) | 0.826 |  |  |
| High-quality embryo |  |  |  |  | 0.452 |  |  |  |  | 0.125 | 0.220 |
| DFI<15% | 2,104 | 0.50 (0.25, 0.75) | Ref |  |  | 195 | 0.50 (0.26, 0.69) | Ref |  |  |  |
| 15%≤DFI<30% | 812 | 0.50 (0.25, 0.75) | 0.94 (0.85, 1.04) | 0.263 |  | 184 | 0.54 (0.24, 0.75) | 1.09 (0.86, 1.39) | 0.480 |  |  |
| DFI≥30% | 179 | 0.50 (0.20, 0.73) | 1.00 (0.81, 1.22) | 0.972 |  | 159 | 0.56 (0.30, 0.75) | 1.24 (0.94, 1.62) | 0.123 |  |  |
| **Blastocyst-Stage** |  |  |  |  |  |  |  |  |  |  |  |
| Blastocyst formation |  |  |  |  | 0.180 |  |  |  |  | 0.819 | 0.836 |
| DFI<15% | 1,655 | 0.50 (0.31, 0.67) | Ref |  |  | 151 | 0.50 (0.33, 0.67) | Ref |  |  |  |
| 15%≤DFI<30% | 589 | 0.45 (0.29, 0.67) | 0.94 (0.86, 1.03) | 0.171 |  | 136 | 0.50 (0.33, 0.67) | 0.99 (0.80, 1.23) | 0.926 |  |  |
| DFI≥30% | 125 | 0.45 (0.25, 0.67) | 0.94 (0.79, 1.13) | 0.507 |  | 115 | 0.50 (0.36, 0.67) | 1.03 (0.81, 1.32) | 0.797 |  |  |
| High-quality blastocyst formation |  |  |  |  | 0.055 |  |  |  |  | 0.327 | 0.140 |
| DFI<15% | 1,655 | 0.13 (0.00, 0.33) | Ref |  |  | 151 | 0.13 (0.00, 0.29) | Ref |  |  |  |
| 15%≤DFI<30% | 589 | 0.13 (0.00, 0.30) | 0.91 (0.80, 1.03) | 0.121 |  | 136 | 0.14 (0.00, 0.33) | 1.10 (0.82, 1.49) | 0.517 |  |  |
| DFI≥30% | 125 | 0.17 (0.00, 0.30) | 0.85 (0.67, 1.07) | 0.171 |  | 115 | 0.20 (0.00, 0.36) | 1.17 (0.85, 1.63) | 0.334 |  |  |

Continuous rates are presented as median (Q1, Q3). Each endpoint across sperm DFI groups was analyzed using either a beta-binomial (BB) or a zero-inflated beta-binomial (ZIBB) regression model, with the final model choice determined by the better fit according to the Akaike Information Criterion (AIC). For the Cleavage-Stage outcomes (fertilization rate, normal fertilization rate, cleavage rate, and high-quality embryo rate), the multivariate models were adjusted for the following covariates: male age, male smoking status, female age, female body mass index, female smoking status, polyendocrine metabolic ovarian syndrome, and the method of fertilization. For the Blastocyst-Stage outcomes (blastocyst formation rate and high-quality blastocyst formation rate), the multivariate models were adjusted for male age, male smoking status, female age, female body mass index, and the method of fertilization. CI, confidence interval; DFI, DNA fragmentation index; OR, odds ratio. **P*<0.05.

| **Supplementary Table S6.** Sensitivity analysis of Blastocyst PGT-A Results Across Sperm DNA Fragmentation Index Groups Categorized by Clinical Threshold | | | | |
| --- | --- | --- | --- | --- |
| **Outcomes** | **Propensity Score Overlap Weighting** | | **DFI testing and oocyte retrieval interval limited to ≤6 months^#^** | |
|  | **Adjusted OR (95% CI)** | ***P* value** | **Adjusted OR (95% CI)** | ***P* value** |
| Whole chromosome aneuploid embryo |  |  |  |  |
| DFI<15% | Ref |  | Ref |  |
| 15%≤DFI<30% | 1.17 (0.75-1.84) | 0.492 | 1.03 (0.85-1.25) | 0.763 |
| DFI≥30% | 1.21 (0.75-1.96) | 0.430 | 1.20 (0.89-1.63) | 0.237 |
| Aneuploid embryo |  |  |  |  |
| DFI<15% | Ref |  | Ref |  |
| 15%≤DFI<30% | 1.13 (0.67-1.89) | 0.647 | 0.92 (0.75-1.14) | 0.455 |
| DFI≥30% | 1.39 (0.81-2.37) | 0.228 | 1.37 (1.01-1.85) | 0.041* |
| Mosaic embryo |  |  |  |  |
| DFI<15% | Ref |  | Ref |  |
| 15%≤DFI<30% | 1.07 (0.66-1.74) | 0.790 | 1.06 (0.85-1.32) | 0.601 |
| DFI≥30% | 0.77 (0.45-1.33) | 0.354 | 0.80 (0.56-1.14) | 0.221 |
| Segmental aneuploid embryo |  |  |  |  |
| DFI<15% | Ref |  | Ref |  |
| 15%≤DFI<30% | 1.10 (0.54-2.26) | 0.793 | 1.14 (0.83-1.57) | 0.409 |
| DFI≥30% | 1.15 (0.54-2.45) | 0.720 | 1.07 (0.65-1.76) | 0.798 |
| ^#^, DFI testing was restricted to within six months before oocyte retrieval. A total of 3,196 blastocysts were included in the final chromosomal analysis. Preimplantation genetic testing for aneuploidies (PGT-A) outcomes across sperm DNA fragmentation index (DFI) groups were analyzed using generalized linear mixed models to account for the random effect of potentially submitting two or more embryos from a single cycle. Covariates adjusted for in both propensity score and multivariable analyses included male age, male smoking status, female age, female body mass index, severe oligoasthenoteratozoospermia, and whether the blastocyst was of high quality. CI, confidence interval; DFI, DNA fragmentation index; OR, odds ratio. **P*<0.05. | | | | |
|  |  |  |  |  |
|  |  |  |  |  |
|  |  |  |  |  |
|  |  |  |  |  |
|  |  |  |  |  |
|  |  |  |  |  |

| **Supplementary Table S7.** Comparative Analysis of Blastocyst PGT-A Results Across Sperm DNA Fragmentation Index Groups Stratified by Female Age | | | | | | | | | |
| --- | --- | --- | --- | --- | --- | --- | --- | --- | --- |
| **Outcomes** | **Female age-stratified subgroups** | | | | | | | | |
|  | **Female age <35** | | | | **Female age ≥35** | | | | **P for interaction** |
|  | **Rate (N=2160)** | **Adjusted OR (95% CI)** | **P value** | **P for trend** | **Rate (N=1414)** | **Adjusted OR (95% CI)** | **P value** | **P for trend** |  |
| Whole chromosome aneuploid embryo |  |  |  | 0.621 |  |  |  | 0.237 | 0.239 |
| DFI<15% | 43.41% (642/1479) | Ref |  |  | 58.48% (486/831) | Ref |  |  |  |
| 15%≤DFI<30% | 45.74% (247/540) | 1.07 (0.84-1.35) | 0.582 |  | 58.61% (211/360) | 0.88 (0.67-1.16) | 0.379 |  |  |
| DFI≥30% | 41.13% (58/141) | 0.76 (0.49-1.18) | 0.228 |  | 69.06% (154/223) | 1.45 (0.99-2.13) | 0.054 |  |  |
| Aneuploid embryo |  |  |  | 0.649 |  |  |  | 0.078 | 0.183 |
| DFI<15% | 16.36% (242/1479) | Ref |  |  | 38.75% (322/831) | Ref |  |  |  |
| 15%≤DFI<30% | 17.04% (92/540) | 0.99 (0.74-1.31) | 0.918 |  | 38.89% (140/360) | 0.88 (0.67-1.17) | 0.378 |  |  |
| DFI≥30% | 16.31% (23/141) | 0.85 (0.50-1.46) | 0.565 |  | 52.02% (116/223) | 1.61 (1.12-2.32) | 0.010* |  |  |
| Mosaic embryo |  |  |  | 0.901 |  |  |  | 0.114 | 0.370 |
| DFI<15% | 26.10% (386/1479) | Ref |  |  | 27.68% (230/831) | Ref |  |  |  |
| 15%≤DFI<30% | 29.26% (158/540) | 1.17 (0.90-1.53) | 0.244 |  | 25.83% (93/360) | 0.85 (0.61-1.17) | 0.314 |  |  |
| DFI≥30% | 22.70% (32/141) | 0.73 (0.44-1.22) | 0.228 |  | 21.52% (48/223) | 0.72 (0.46-1.12) | 0.147 |  |  |
| Segmental aneuploid embryo |  |  |  | 0.940 |  |  |  | 0.887 | 0.820 |
| DFI<15% | 9.60% (142/1479) | Ref |  |  | 9.75% (81/831) | Ref |  |  |  |
| 15%≤DFI<30% | 9.81% (53/540) | 0.96 (0.64-1.43) | 0.846 |  | 11.67% (42/360) | 1.10 (0.69-1.75) | 0.677 |  |  |
| DFI≥30% | 11.35% (16/141) | 1.10 (0.55-2.22) | 0.787 |  | 10.31% (23/223) | 0.99 (0.53-1.87) | 0.987 |  |  |
| Preimplantation genetic testing for aneuploidies (PGT-A) outcomes across sperm DNA fragmentation index (DFI) groups were analyzed using generalized linear mixed models to account for the random effect of potentially submitting two or more embryos from a single cycle. The multivariate model was adjusted for male age, male smoking status, female age, female body mass index, severe oligoasthenoteratozoospermia, and whether the blastocyst was of high quality. CI, confidence interval; DFI, DNA fragmentation index; OR, odds ratio. **P*<0.05. | | | | | | | | | |

| **Supplementary Table S8.** Comparative Analysis of Blastocyst PGT-A Results Across Sperm DNA Fragmentation Index Groups Stratified by Male Age | | | | | | | | | |
| --- | --- | --- | --- | --- | --- | --- | --- | --- | --- |
| **Outcomes** | **Male age-stratified subgroups** | | | | | | | | |
|  | **Male age <40** | | | | **Male age ≥40** | | | | ***P* for interaction** |
|  | **Rate (N=2829)** | **Adjusted OR (95% CI)** | ***P* value** | ***P* for trend** | **Rate (N=745)** | **Adjusted OR (95% CI)** | ***P* value** | ***P* for trend** |  |
| Whole chromosome aneuploid embryo |  |  |  | 0.675 |  |  |  | 0.174 | 0.005** |
| DFI<15% | 46.44% (921/1983) | Ref |  |  | 63.30% (207/327) | Ref |  |  |  |
| 15%≤DFI<30% | 45.83% (308/672) | 0.95 (0.77-1.16) | 0.598 |  | 65.79% (150/228) | 1.28 (0.86-1.91) | 0.218 |  |  |
| DFI≥30% | 48.85% (85/174) | 0.99 (0.68-1.43) | 0.949 |  | 66.84% (127/190) | 1.35 (0.84-2.17) | 0.213 |  |  |
| Aneuploid embryo |  |  |  | 0.961 |  |  |  | 0.139 | 0.525 |
| DFI<15% | 21.43% (425/1983) | Ref |  |  | 42.51% (139/327) | Ref |  |  |  |
| 15%≤DFI<30% | 19.94% (134/672) | 0.89 (0.70-1.12) | 0.322 |  | 42.98% (98/228) | 1.13 (0.78-1.65) | 0.510 |  |  |
| DFI≥30% | 27.01% (47/174) | 1.22 (0.81-1.82) | 0.338 |  | 48.42% (92/190) | 1.42 (0.91-2.21) | 0.124 |  |  |
| Mosaic embryo |  |  |  | 0.722 |  |  |  | 0.105 | 0.657 |
| DFI<15% | 26.17% (519/1983) | Ref |  |  | 29.66% (97/327) | Ref |  |  |  |
| 15%≤DFI<30% | 27.98% (188/672) | 1.07 (0.85-1.35) | 0.574 |  | 27.63% (63/228) | 0.88 (0.56-1.37) | 0.568 |  |  |
| DFI≥30% | 23.56% (41/174) | 0.81 (0.53-1.26) | 0.350 |  | 20.53% (39/190) | 0.62 (0.35-1.09) | 0.099 |  |  |
| Segmental aneuploid embryo |  |  |  | 0.941 |  |  |  | 0.612 | 0.083 |
| DFI<15% | 9.43% (187/1983) | Ref |  |  | 11.01% (36/327) | Ref |  |  |  |
| 15%≤DFI<30% | 9.52% (64/672) | 0.97 (0.68-1.40) | 0.881 |  | 13.60% (31/228) | 1.27 (0.73-2.20) | 0.396 |  |  |
| DFI≥30% | 9.77% (17/174) | 1.02 (0.54-1.94) | 0.951 |  | 11.58% (22/190) | 1.13 (0.57-2.23) | 0.719 |  |  |
| Preimplantation genetic testing for aneuploidies (PGT-A) outcomes across sperm DNA fragmentation index (DFI) groups were analyzed using generalized linear mixed models to account for the random effect of potentially submitting two or more embryos from a single cycle. The multivariate model was adjusted for male age, male smoking status, female age, female body mass index, severe oligoasthenoteratozoospermia, and whether the blastocyst was of high quality. CI, confidence interval; DFI, DNA fragmentation index; OR, odds ratio. ***P*<0.01. | | | | | | | | | |
|  |  |  |  |  |  |  |  |  |  |
|  |  |  |  |  |  |  |  |  |  |
|  |  |  |  |  |  |  |  |  |  |

| **Supplementary Table S9.** Comparative Analysis of Blastocyst PGT-A Results Across Sperm DNA Fragmentation Index Groups Stratified by Severe Oligoasthenoteratozoospermia Status | | | | | | | | | |
| --- | --- | --- | --- | --- | --- | --- | --- | --- | --- |
| **Outcomes** | **Severe oligoasthenoteratospermia status-stratified subgroups** | | | | | | | | |
|  | **No** | | | | **Yes** | | | | ***P* for interaction** |
|  | **Rate (N=3054)** | **Adjusted OR (95% CI)** | ***P* value** | ***P* for trend** | **Rate (N=520)** | **Adjusted OR (95% CI)** | ***P* value** | ***P* for trend** |  |
| Whole chromosome aneuploid embryo |  |  |  | 0.976 |  |  |  | 0.176 | 0.314 |
| DFI<15% | 49.37% (1,050/2127) | Ref |  |  | 42.62% (78/183) | Ref |  |  |  |
| 15%≤DFI<30% | 50.96% (370/726) | 0.94 (0.77-1.16) | 0.579 |  | 50.57% (88/174) | 1.34 (0.86-2.08) | 0.191 |  |  |
| DFI≥30% | 59.70% (120/201) | 1.10 (0.77-1.59) | 0.595 |  | 56.44% (92/163) | 1.38 (0.84-2.27) | 0.211 |  |  |
| Aneuploid embryo |  |  |  | 0.956 |  |  |  | 0.022* | 0.121 |
| DFI<15% | 24.96% (531/2127) | Ref |  |  | 18.03% (33/183) | Ref |  |  |  |
| 15%≤DFI<30% | 26.45% (192/726) | 0.88 (0.71-1.10) | 0.266 |  | 22.99% (40/174) | 1.39 (0.79-2.43) | 0.256 |  |  |
| DFI≥30% | 39.30% (79/201) | 1.20 (0.84-1.71) | 0.315 |  | 36.81% (60/163) | 1.99 (1.11-3.57) | 0.022* |  |  |
| Mosaic embryo |  |  |  | 0.267 |  |  |  | 0.593 | 0.430 |
| DFI<15% | 26.85% (571/2127) | Ref |  |  | 24.59% (45/183) | Ref |  |  |  |
| 15%≤DFI<30% | 28.65% (208/726) | 1.05 (0.84-1.32) | 0.673 |  | 24.71% (43/174) | 0.95 (0.57-1.59) | 0.853 |  |  |
| DFI≥30% | 21.39% (43/201) | 0.67 (0.44-1.02) | 0.062 |  | 22.70% (37/163) | 0.84 (0.47-1.53) | 0.574 |  |  |
| Segmental aneuploid embryo |  |  |  | 0.847 |  |  |  | 0.587 | 0.958 |
| DFI<15% | 9.64% (205/2127) | Ref |  |  | 9.84% (18/183) | Ref |  |  |  |
| 15%≤DFI<30% | 10.33% (75/726) | 1.01 (0.73-1.41) | 0.936 |  | 11.49% (20/174) | 1.29 (0.61-2.73) | 0.506 |  |  |
| DFI≥30% | 10.95% (22/201) | 1.08 (0.61-1.92) | 0.784 |  | 10.43% (17/163) | 1.22 (0.53-2.85) | 0.638 |  |  |
| Preimplantation genetic testing for aneuploidies (PGT-A) outcomes across sperm DNA fragmentation index (DFI) groups were analyzed using generalized linear mixed models to account for the random effect of potentially submitting two or more embryos from a single cycle. The multivariate model was adjusted for male age, male smoking status, female age, female body mass index, and whether the blastocyst was of high quality. CI, confidence interval; DFI, DNA fragmentation index; OR, odds ratio. **P*<0.05. | | | | | | | | | |
|  |  |  |  |  |  |  |  |  |  |
|  |  |  |  |  |  |  |  |  |  |
|  |  |  |  |  |  |  |  |  |  |

| **Supplementary Table S10.** Comparative Analysis of Blastocyst PGT-A Results Across Sperm DNA Fragmentation Index Groups Stratified by Blastocyst Quallity | | | | | | | | | |
| --- | --- | --- | --- | --- | --- | --- | --- | --- | --- |
| **Outcomes** | **High-quality blastocyst-stratified subgroups** | | | | | | | | |
|  | **No** | | | | **Yes** | | | | ***P* for interaction** |
|  | **Rate (N=1081)** | **Adjusted OR (95% CI)** | ***P* value** | ***P* for trend** | **Rate (N=2493)** | **Adjusted OR (95% CI)** | ***P* value** | ***P* for trend** |  |
| Whole chromosome aneuploid embryo |  |  |  | 0.274 |  |  |  | 0.991 | 0.261 |
| DFI<15% | 64.18% (421/656) | Ref |  |  | 42.74% (707/1654) | Ref |  |  |  |
| 15%≤DFI<30% | 68.95% (211/306) | 1.23 (0.89-1.70) | 0.208 |  | 41.58% (247/594) | 0.91 (0.73-1.12) | 0.360 |  |  |
| DFI≥30% | 69.75% (83/119) | 1.18 (0.71-1.94) | 0.526 |  | 52.65% (129/245) | 1.11 (0.80-1.56) | 0.528 |  |  |
| Aneuploid embryo |  |  |  | 0.065 |  |  |  | 0.969 | 0.503 |
| DFI<15% | 35.06% (230/656) | Ref |  |  | 20.19% (334/1654) | Ref |  |  |  |
| 15%≤DFI<30% | 39.87% (122/306) | 1.17 (0.86-1.58) | 0.318 |  | 18.52% (110/594) | 0.79 (0.61-1.02) | 0.073 |  |  |
| DFI≥30% | 47.90% (57/119) | 1.53 (0.97-2.41) | 0.069 |  | 33.47% (82/245) | 1.20 (0.84-1.72) | 0.315 |  |  |
| Mosaic embryo |  |  |  | 0.290 |  |  |  | 0.547 | 0.719 |
| DFI<15% | 35.98% (236/656) | Ref |  |  | 22.97% (380/1654) | Ref |  |  |  |
| 15%≤DFI<30% | 36.27% (111/306) | 1.03 (0.75-1.41) | 0.859 |  | 23.57% (140/594) | 1.04 (0.81-1.34) | 0.734 |  |  |
| DFI≥30% | 27.73% (33/119) | 0.68 (0.41-1.12) | 0.130 |  | 19.18% (47/245) | 0.80 (0.53-1.21) | 0.295 |  |  |
| Segmental aneuploid embryo |  |  |  | 0.084 |  |  |  | 0.349 | <0.001*** |
| DFI<15% | 13.87% (91/656) | Ref |  |  | 7.98% (132/1654) | Ref |  |  |  |
| 15%≤DFI<30% | 16.99% (52/306) | 1.28 (0.82-2.01) | 0.277 |  | 7.24% (43/594) | 0.92 (0.57-1.48) | 0.731 |  |  |
| DFI≥30% | 21.01% (25/119) | 1.73 (0.89-3.34) | 0.105 |  | 5.71% (14/245) | 0.66 (0.30-1.46) | 0.306 |  |  |
| Preimplantation genetic testing for aneuploidies (PGT-A) outcomes across sperm DNA fragmentation index (DFI) groups were analyzed using generalized linear mixed models to account for the random effect of potentially submitting two or more embryos from a single cycle. The multivariate model was adjusted for male age, male smoking status, female age, female body mass index, and severe oligoasthenoteratozoospermia. CI, confidence interval; DFI, DNA fragmentation index; OR, odds ratio. ****P*<0.001. | | | | | | | | | |
|  |  |  |  |  |  |  |  |  |  |
|  |  |  |  |  |  |  |  |  |  |
|  |  |  |  |  |  |  |  |  |  |
