# Supplementary material for: High sperm DNA fragmentation is associated with increased embryonic chromosomal abnormality: a retrospective study of 8 years involving 5637 couples undergoing ART
Source: Hum Reprod Open. 2026 Sep 16;2026(4):hoag082. doi: 10.1093/hropen/hoag082 (PMC13658023; doi:10.1093/hropen/hoag082)
Supplement: Supplementary file 1 [file hoag082_supplementary_data.zip › HRO-26-0406-R2-SuppFigs1and2.docx]

**Supplementary Figure S1. Association between sperm DNA fragmentation index and conventional semen quality parameters.**

DNA fragmentation index (DFI) was analyzed as a continuous variable using restricted cubic spline models. The plots display the estimated marginal effects (regression coefficients) of DFI on each semen parameter, representing the predicted change in outcome per 1% increase in DFI. Adjusted factors included male age, male body mass index and male smoking status. Shaded areas indicate 95% confidence intervals, *P*-values for overall association and non-linearity are indicated for each model.

**Supplementary Figure S2. Association between sperm DNA fragmentation index and embryo development parameters.**

DNA fragmentation index (DFI) was modeled as a continuous variable using restricted cubic splines (RCS). Plots depict the estimated marginal effects of DFI on each outcome, representing the predicted change per 1% increase in DFI. Shaded areas denote 95% confidence intervals, P-values for overall association and non-linearity are shown for each model. **Cleavage-stage outcomes** (fertilization rate, normal fertilization rate, cleavage rate, and high-quality embryo rate) were adjusted for female age, male age, maternal and paternal smoking status, female body mass index, polyendocrine metabolic ovarian syndrome (PMOS), severe oligoasthenoteratozoospermia (SOAT), and fertilization method. **Blastocyst-stage outcomes** (blastocyst formation rate and high-quality blastocyst formation rate) were adjusted for female age, male age, male smoking status, female body mass index, SOAT, and fertilization method.
